# Post-genomic clinical trials: the perspective of ACGT

**DOI:** 10.3332/eCMS.2008.66

**Published:** 2008-01-21

**Authors:** N Graf, C Desmedt, F Buffa, D Kafetzopoulos, N Forgó, R Kollek, A Hoppe, G Stamatakos, M Tsiknakis

**Affiliations:** 1 University Hospital of Saarland Paediatric Haematology and Oncology, D-66421 Homburg, Germany; 2 Institut Jules Bordet, B-1000 Bruxelles, Belgium; 3 Weatherall Institute of Molecular Medicine, Growth Factors Group Cancer Research UK, University of Oxford, Oxford, OX3 9DS, UK; 4 Foundation for Research & Technology-Hellas, GR-71110 Heraklion, Greece; 5 Institut für Rechtsinformatik, Leibniz Universität Hannover, Juristische Fakultät, D-30167 Hannover, Germany; 6 Universität Hamburg FSP BIOGUM, D - 20251 Hamburg, Germany; 7 Institute of Communication and Computer Systems, School of Electrical and Computer Engineering, National Technical University of Athens, GR-157 80 Zografos, Greece

## Introduction

Cancer is a complex heterogeneous disease developing from integrated actions of multiple genetic and environmental factors through dynamic epigenetic and molecular regulatory mechanisms. One can find the complexity of cancer at the physiological cellular tissue and organ levels. There are interactions between tumours and their micro-environments, promoting their growth survival and the occurrence of distant metastasis [1]. However, the molecular mechanisms underlying these processes are poorly understood. It is reasonable to think that each cancer cell within a tumour might originate through different cancer-specific developmental mechanisms and mutations in distinct genes. There is increasing evidence that cancer initiation results from accumulative oncogenic mutations in long-lived stem cells or their immediate progenitor [2]. It is believed that signalling pathways, which regulate self-renewal in normal stem cells are deregulated in cancer-initiating cells, resulting in uncontrolled expansion and aberrant differentiation and formation of tumours with a heterogeneous phenotype [3]. The molecular changes within the tumour cells are followed by modification of the surrounding micro-environment.

During the last few years the ‘omics’ revolution has dramatically increased the amount of data available for characterizing intracellular events. On the methodological level, most important for this development are differential gene expression analysis for recording mRNA concentration profiles and proteomics for providing data on protein abundance [4,5]. Soon after microarrays were introduced many researchers realized that the technique could be used to identify biologic markers associated with disease [6] and even with subclasses of disease [7–10]. As a result, a lot of patterns of expression were found that could be used to classify molecular subtypes of tumours [11] and predict the outcome [12–14] and response to treatment [15–17].

But the initial enthusiasm for the application of microarray technology was tempered by the publication of several studies, reporting contradictory results on the analysis of the same RNA samples hybridized on different microarray platforms. Scepticism arose regarding the reliability and the reproducibility of this technique. Most of the discrepancies were attributed to inconsistent sequence fidelity and annotation, low specificity of the spotted cDNA microarrays, lack of probe specificity for different isoforms or differences in the hybridization conditions, fluorescence measurement, normalization strategies and analytical algorithms applied [18–23]. One main source of the problem was also shown to be the small number of samples that were used to generate the gene lists of these experiments [24]. In view of these concerns raised on one hand and the great potential of this technology for tailored medicine on the other hand, the US Food and Drug Administration launched the Microarray Quality Control (MAQC) project, involving 137 participants from 51 academic and industry partners to systemically address the technical reproducibility of microarray measurements within and between laboratories as well as across different microarray platforms. The results derived from this collaborative effort showed that the microarray measurements are highly reproducible within and across different microarray platforms, and that microarray technologies are sufficiently reliable to be used for clinical and regulatory purposes [25].

Currently, the main focus is on interlinking the various data sources generated by high-throughput array technologies [26]. There are two different ways of doing so: the systems biology approach and the biological networks. The approach of systems biological studies is to combine information from molecular biology genetics and epidemiology with comprehensive mathematical models to study how gene–gene interactions, gene–environment interactions and protein–protein interactions act together to cause disease [27]. On the other hand, the biological networks, also known as pathways, begin with the knowledge of known genes and proteins in an organism. In the next step, changes between normal and pathological systems are measured using either high-throughput techniques, such as gene expression microarrays for mRNAs or proteomics methods for protein concentrations [28,29]. A crucial part of this process is to model the inherent stochastic nature of the system [30–32]. This information on functional molecular interactions [33]—known as pathway databases—enriches our understanding of cellular systems [34]. Although the biological networks and systems biology approaches are very similar, biological networks are based more on biochemical reactions and signalling interactions among active proteins. This dynamic network is called the ‘interactome’. Hence, they rely more heavily on systemic network analysis, and other data-mining techniques compared with systems biology, which emphasizes statistical learning [35].

Recently, systems biological research has been providing a framework for such integration. Various groups have applied network analysis to gene data sets associated with cancer. Jonsson and Bates reported very recently that proteins associated with cancer show an increased number of interacting partners in the interactome [36]. Wachi *et al* specifically investigated the role of the interactome of genes differentially regulated in lung cancer [37]. Tuck and colleagues analysed transcriptional regulatory networks consisting of transcription factors and their target proteins [38]. Genes differentially regulated between acute myeloid leukaemia and acute lymphoblastic leukaemia were significantly closer in the network as compared to randomly generated gene lists. The analogous result was observed for genes differentially regulated in breast cancer patients. On a more general level Xu and Li showed that disease-associated genes as listed in the OMIM database [39] tend to interact with other disease-associated genes [40].

Advancing Clinico-Genomic Trials (**ACGT**), a project funded by the European Commission in the Sixth Framework Programme, goes far beyond the systems biologic approach and the biological network by the addition of integrating clinical data. The ultimate objective of the **ACGT** project is the provision of a unified technological infrastructure, which will facilitate the seamless and secure access and analysis of multi-level clinical and genomic data enriched with high-performing knowledge discovery operations and services. By doing so, it is expected that the influence of genetic variation in oncogenesis will be revealed, the molecular classification of cancer and the development of individualized therapies will be promoted, and finally, the *in silico* tumour growth and therapy response will be realistically and reliably modelled. Achieving these goals, **ACGT** will not only secure the advancement of clinico-genomic trials but will also achieve an expandable environment to other studies’ technologies and tools.

**The** vision of **ACGT** is to become a pan-European voluntary network connecting individuals and institutions to enable the sharing of data and tools and thereby creating a European-wide web of cancer clinical research. In achieving this objective, **ACGT** will:

1. deliver a European Biomedical GRID infrastructure, offering seamless mediation services for sharing data and data-processing methods and tools;
2. deliver advanced security tools, including anonymization and pseudonymization of personal data according to European legal and ethical regulations;
3. develop an ACGT Master Ontology and use standard clinical and genomic ontologies and metadata for the semantic integration of heterogeneous data (clinical imaging genomic proteomic metabolomic and other as well as open source data from the web);
4. develop an Ontology-Based Trial builder for helping to easily set up new clinico-genomic trials to collect clinical research and administrative data and to put researchers in the position to perform cross-trial analysis;
5. deliver data-mining services in order to support and improve complex knowledge discovery processes.

The technological platform of **ACGT** will be validated in the concrete setting of **clinical trials** on **Cancer**. Pilot trials have been developed based on the presence of clear research objectives, raising the need to integrate data at all levels. This integrative view underlies the development of clinico-genomic models, showing that the combination of biomarkers and clinical factors are most relevant in terms of statistical fit and also more practically in terms of cross-validation predictive accuracy [41].

## Clinical trials in cancer

In Europe, there are a lot of ongoing clinical trials and studies related to cancer. These trials will guarantee the best available treatment for patients with cancer and will provide the highest level of quality control if done according to GCP criteria [42]. However, amongst the different hospitals involved, there is heterogeneity in the way patients’ data are documented. The most important parts of data management systems in clinical trials are the Case Report Forms (CRFs), which are designed to collect the required research and administrative data and the trial database to store these data. In many multi-centre trials, paper-based CRFs are still used today. From the participating hospitals, thousands of CRFs are sent to a central data facility where the data are entered into a trial database. This is very time consuming and error prone. Often, the clinical trial databases are in-house developments that have to be implemented from scratch for each new trial [43]. Today, the preferable systems are web-based remote data-entry systems, where the data are captured at the participating site and transferred electronically to the trial central data facility. Most of these management systems allow designing the trial and especially creating electronic CRFs by the trial chairmen without any informatics skills. But none of these systems use an ontology, resulting in clinical trial databases that do not comprise comprehensive metadata, and that are not standardized. It is highly problematic to use such data for further research analysis. These difficulties and limitations are pronounced in efforts to extend national clinical trials to international ones.

It is obvious that current clinical trial methodologies are not exploiting the technological advances offered. In **ACGT**, an ontology-based trial management system will be developed to enable trial chairmen to set up interoperable clinical data management systems. The system is called the ‘Ontology-based Trial Management System of ACGT’ (ObTiMA). ObTiMA consists of three parts:

1. Trial Builder.
  - Trial Outline Builder (TOB).
    - Including a graphical schema of the trial.
  - CRF Creator (CC).
2. Repository.
3. Patient Data Management System (PDMS).

The Trial Builder is primarily used to build a new trial. The user will be guided by a Master Protocol for clinical trials to write the Trial Protocol to build a graphical schema of the trial and to create all CRFs that are needed for the trial. All legal and ethical requirements will be considered during this process and appropriate solutions provided. ObTiMA maintains and manages the planning preparation performance and reporting of clinical trials with emphasis on keeping up-to-date contact information for participants and tracking deadlines and milestones such as those for regulatory approval or the issue of progress reports.

By creating new CRFs, the database for the trial will be automatically generated and is always ontology based, including comprehensive metadata. The advantage of integrating an ontology in the design process is the built-in semantic interoperability. Data collected with this system can be seamlessly integrated into a data integration framework like ACGT, using the same reference ontology. The integration of the ontology in the process of creating CRFs will automatically help to maintain the ontology and enhance the use of ontologies in clinical trials in the future. The ACGT Trial Builder will support a modular concept. According to the modularity, there is the need for a repository for trials and CRFs for reuse. The PDMS is the data management system of the trial used by participants of a trial via remote data entry (RDE). ObTiMA will be a component-based extendable application.

Today, it is recognized that the key to individualizing treatment for cancer lies in finding a way to quickly ‘translate’ the discoveries about human genetics made by laboratory scientists into tools that physicians can use in making decisions about the best way to treat patients. This area of medicine that links basic laboratory study to clinical data, including the treatment of patients, is called translational research and is promoted by clinico-genomic trials running in ACGT. These clinico-genomic trials are scenario based and driven by clinicians. Today, two main clinico-genomic trials and an *in silico* experiment are interconnected within the ACGT project. The realization of these trials will act as benchmark references for the development and assessment of the ACGT technology.

## Clinicogenomic trials

1. The first clinico-genomic trial focuses on **breast cancer** and uses gene-expression profiling based on microarrays as well as genotyping technology to identify predictive markers of response/resistance for anthracyclines chemotherapy.
2. The second trial focuses on **paediatric nephroblastoma** (Wilms tumour) and addresses the treatment of these patients according to well-defined risk groups in order to achieve highest cure rates to decrease the frequency and intensity of acute and late toxicity and to minimize the cost of therapy. The main objective of this trial is to explore a pattern of auto-antibodies against nephroblastoma-specific antigens as a new diagnostic and prognostic tool for the more individualized stratification of treatment.

**In silico *oncology***
3. The *in silico* oncology focuses on the development and evaluation of tumour growth and response to treatment. The aim is to develop an ‘oncosimulator’ and evaluate the reliability of *in silico* modelling as a tool for assessing alternative cancer treatment strategies especially in the case of combining and utilizing mixed clinical imaging and genomic/genetic information and data.

## Breast cancer

Breast cancer (BC) is the commonest cancer in women in the world in both industrialized and developing countries. Over a million, women will be diagnosed with breast cancer worldwide in 2004 [44]. More than 40,000 women will die this year of metastatic breast cancer in the United States alone, and more than 200,000 new cases of cancer will be detected [45]. The mortality rate around the world especially in developing countries is much higher, making breast cancer a significant public health problem.

Breast cancer is both genetically and histopathologically heterogeneous, and the mechanisms underling breast cancer development remains largely unknown. Breast cancer patients diagnosed with the same stage of disease often have remarkably different responses to therapy and overall outcome. Even with the strongest prognostic indicators, such as lymph node status, oestrogen receptor expression and histological grade, it is not possible to accurately classify breast tumours according to their clinical behaviour. Therefore, most patients are routinely treated with an adjuvant chemotherapy or hormonal therapy to reduce the risk of distant metastases. However, 70–80% of patients receiving this aggressive treatment would have survived without it, and therefore suffered unnecessarily from accompanying side effects [46]. A molecular marker with predictive power for breast cancer is going to benefit almost three out of four women that receive aggressive chemotherapy treatment although they would have survived without it.

Much progress has been made over the past decades in our understanding of the epidemiology clinical course and basic biology of breast cancer. Identified risk factors include:

1. Family history (genetics). Identified gene mutations represent a tiny fraction of all breast cancers, much less than 10% overall. But, if present, they confer considerable lifetime risk compared to the general population.
2. Reproductive and hormonal life, for example early menarche, no pregnancy or late age at first birth, late menopause hormonal factors, such as high levels of free oestrogen, long-term use of oral contraceptives or menopausal hormone replacement or other factors that increase life-time exposure to oestrogen.
3. Lifestyle, particularly diet and exposures to carcinogenic agents.

The heterogeneity of both the disease and the causal factors makes the clinical assessment difficult. This difficulty is mainly attributable to the first 5–10 years since the long-term outcome is rather predictable after this time. The standard markers for the assessment are morphological (size infiltration, lymph node metastasis, etc) and molecular (oestrogen and progesterone receptors status and her2/Neu). Although very useful for the clinicians, they are ‘subjects to subjectivity’ and surely not good enough to make the therapeutic decision accurate. Global expression analysis using microarrays now offers unprecedented opportunities to obtain molecular signatures of the state of activity of diseased cells and patient samples. This groundbreaking approach to studying cancer promises to provide a better understanding of the underlying mechanism for tumorgenesis, more accurate diagnosis, more comprehensive prognosis and more effective therapeutic interventions. Given the clinical heterogeneity of breast cancer microarrays it is an ideal tool to establish a more accurate classification [47]. But the question of whether these signatures are a better prognostic tool on adjuvant decision making than traditional clinico/pathological factors is still unanswered.

Using the preoperative approach combined with microarray and proteomics analysis of pre- and post-treatment biopsies, the TOP and FRAGRANCE multi-centre trials both coordinated by the Jules Bordet Institute (ACGT partner) aim to identify novel molecular markers/signatures predictive of response/resistance to anthracycline-based chemotherapy and endocrine therapy, respectively. Currently, TRANSBIG, a newly created translational research network affiliated with the Breast International Group (BIG), launched an innovative worldwide clinical trial, aiming to evaluate the prognostic value of the 70-gene signature identified by the Amsterdam group [14]. The MINDACT trial will test the hypothesis that gene classification based on the gene expression profiles of adjuvant breast cancer patients may allow for significant reduction in adjuvant chemotherapy prescription compared with the traditional methods.

The management of metastatic breast cancer has also evolved and improved over the last few decades [48]. Today, therapy decision making involves the consideration of many clinical parameters. Making the correct pathological diagnosis is always preferred before the initiation of treatment of the cancer patient, because it would facilitate the individualization of treatment and also because of the fact that cancer tends to become more aggressive as time passes by. Using standard pathological techniques, it is estimated that up to 5–10% of all tumours may actually be misclassified [49, 50].

**Figure 1:**
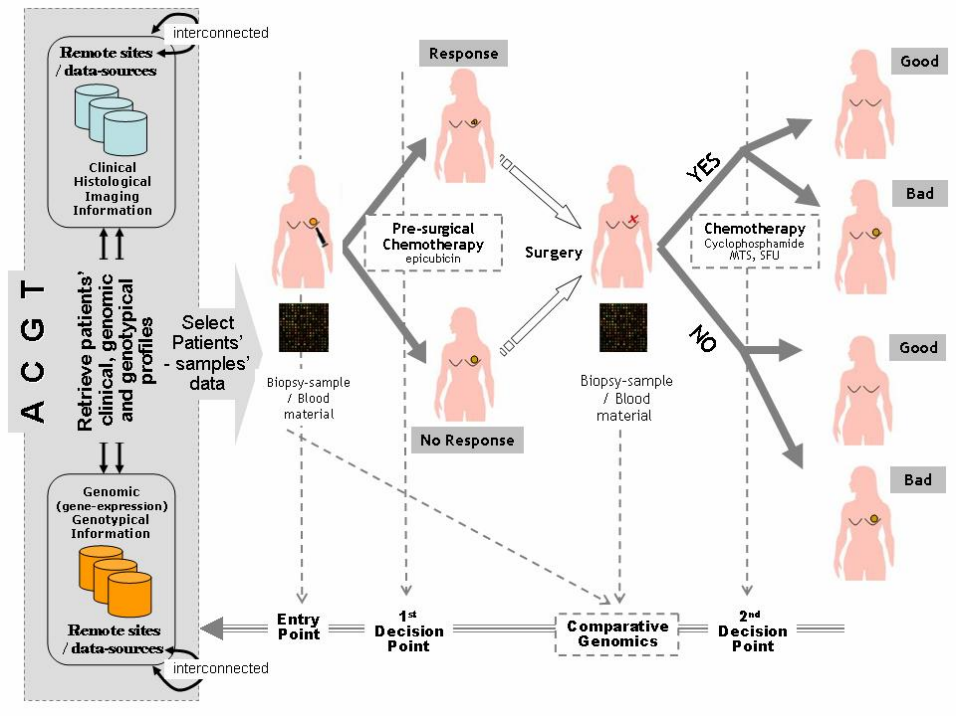
Breast cancer clinico-genomic trials—‘entry point’ of the clinico-genomic trial is realized by access to the ACGT environment, integrating relevant data sources from remote sites in order to retrieve patients’ data that meet specified clinico-genomic/genotypic profiles ‘first and second decision points’ are also supported by ACGT, induction and assessment of pre- and post-surgical treatment and molecular signatures for the prognosis classification of breast cancer patients (a line for knowledge-discovery and clinical decision-making research), ‘molecular analysis’ is also supported by ACGT in order to ease exploration and induction of fundamental molecular knowledge (gene expression profiling, comparative genomics, proteinomics, etc).

There are two basic scenarios foreseen for the realization of the breast cancer clinico-genomic trials:

1. *BC-scenario 1 – Chemotherapeutic treatment:* a chemotherapy assessment scenario addressing the treatment of breast cancer patients based on the molecular characterization of pre- and meta-surgical chemotherapy response. The goal is to induce breast cancer chemotherapeutic treatment strategies and drug administration alternatives on the basis of patients’ individual clinico-genomic profiles. Furthermore, an additional aim is to form and validate respective clinico-genomic breast cancer treatment guidelines and drug-administration protocols.
2. *BC-scenario 2 – Decision making*: a decision-making scenario addressing the operational workflows involved in the course of managing breast cancer patients, i.e. identification of relative guidelines and best-practice protocols being induced and validated by the aforementioned BC-Scenario 1 above. In other words, it presents a scenario of how the outcome and results of clinico-genomic trials are utilized in the course of normal clinical decision making. The aim is to form evaluate and validate the involved decision-making processes as realized and offered by the integrated ACGT environment and platform (Figure 1).

## Nephroblastoma

Wilms tumour (nephroblastoma) is the most common malignant renal tumour in children. Dramatic improvements in survival have occurred as the result of advances in anaesthetic and surgical management, irradiation and chemotherapy and the enrolment of nearly all patients with this disease in clinical trials for more than 30 years. Today, treatments are based on several multi-centre trials and studies conducted by the International Society of Paediatric Oncology (SIOP) in Europe and Children’s Oncology Group (COG) in Northern America. The main objectives of these trials and studies are to treat patients according to well-defined risk groups in order to achieve highest cure rates, to decrease the frequency and intensity of acute and late toxicity, and to minimize the cost of therapy. In that way, the SIOP trials and studies largely focus on the issue of preoperative therapy. The concept of neoadjuvant chemotherapy plays an important role in the treatment for most paediatric solid tumours today. The complete surgical removal of a shrunken tumour is facilitated, and mutilation caused by surgical procedures is minimized or avoided and micro-metastases not visible at diagnosis are treated as early as possible. Besides, the response to treatment can be measured individually by tumour volume reduction and/or percentage of therapy-induced necrosis in the histological specimen.

The International Society of Paediatric Oncology enrolled children with Wilms tumour in six studies up to now (SIOP 1, SIOP 2, SIOP 5, SIOP 6, SIOP 9, SIOP 93-01). The seventh trial and study (SIOP 2001) started in 2002 and is still recruiting patients. A review of the SIOP studies is given by Graf *et al* [51]. Since 1994, more than 1500 patients with a kidney tumour are enrolled in the SIOP studies and trials in Germany. Today, more than 90% of patients with Wilms tumour can be cured, as shown for stage I patients in the trial SIOP 93-01 [52].

The challenges and the main motivation for deploying the SIOP nephroblastoma trial within **ACGT** are:

1. *The distributed nature of the participating clinical sites*: there are more than 200 hospitals treating children with nephroblastoma according to the same SIOP protocol. These hospitals are mainly located around Europe and few are elsewhere in the world. There is a clear need to seamlessly integrate data from all these sites.
2. *The fact that microarray-based research is still not included in any nephroblastoma trial*: although both the SIOP and the COG are promoting the use of microarray analysis to enhance clinical trials, there is a need to integrate clinico-genomic data in order to investigate prognostic factors and assess the potential of individualized therapy. The **ACGT** promotes this integration and provides the necessary analytic tools and standards for clinical trials.
3. *Heterogeneity of data*: data collected are: images of the tumour at different time points related to the treatment, information about treatment itself (surgery, chemotherapy and irradiation), data regarding acute toxicity and late effects, information about relapse and outcome, and microarray data and other molecular genetic data for a limited set of patients.

**The ACGT** will promote the integration of all this information to facilitate further molecular analysis access to tissue banks, provide the necessary analytic tools and allow clinicians to efficiently analyse data that are presently communicated by mail or maintained in flat text files at various remote clinical sites.

In the SIOP trials, the diagnosis is done by imaging studies alone before starting preoperative chemotherapy. A definitive diagnosis is available after histological proof after surgery of the tumour. As a disadvantage, 1% of children receive chemotherapy whilst having a benign disease. In this respect, the ACGT nephroblastoma trial is based on one scenario that is highly important for helping to assure the correct diagnosis before starting any kind of treatment.

## Wilms-scenario: tumour-specific antigens

**Figure 2:**
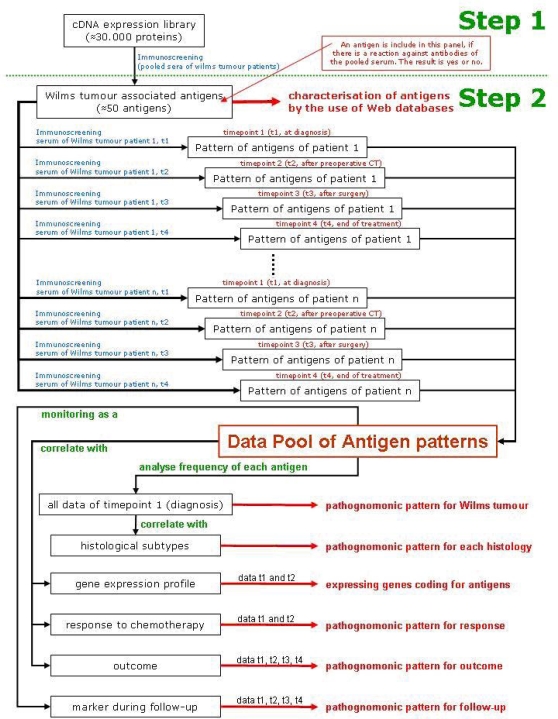
Schematic description of the scenario

Immunogenic tumour-associated antigens have been reported for a variety of malignant tumours, including brain tumours and prostate, lung and colon cancer [53 54]. In a first step, immunogenic Wilms tumour-associated antigens will be identified by immuno-screening of a cDNA expression library. This first step will identify those antigens that show reactivity against serum antibodies of patients with Wilms tumour and not with healthy individuals. They will be characterized using web databases (Table 1). Only these antigens will be used in step 2 of the scenario, where serum from a specific patient will be tested against these newly identified Wilms tumour antigens. As a result, a specific pattern of antigens will be found in each patient and correlated to the histological subtype of the tumour, the gene expression profiling of the tumour, the response to chemotherapy and the outcome of the patient (Figure 2).

The pattern of the identified antigens will contribute to answering key questions about the humeral immune response in Wilms tumour patients:

1. Are Wilms tumours associated with frequent antibody response?
2. Is there a complex and/or specific antibody response?
3. Is this response associated with specific genetic features, like gene amplifications or DNA losses?
4. Do these immunogenic antigens share common features like specific sequence motives?
5. Does the seroreactivity pattern allow early identification of Wilms tumours and also their histological subtypes?
6. Does the seroreactivity pattern represent a prognostic marker for Wilms tumours in respect to chemotherapeutic response and/or outcome?

## In silico *oncology*

Currently, cancer treatment decision and planning is based to a large extent on the disease behaviour of the statistically ‘mean’ patient rather than on the behaviour of each individual case. Therefore, critical details of the particular patient’s tumour biology, such as gene expression profile in conjunction with imaging data, are largely ignored. To alleviate this deficiency, ACGT will develop patient individualized tumour growth and tumour and normal tissue response-simulation models concerning breast cancer and nephroblastoma. Furthermore, the *in silico* application will demonstrate the flexibility of the **ACGT** environment and its potential to become an European platform for both conducting clinical trials and implementing demanding applications. The *in silico* oncology systems under development will serve as basic research tools in the cancer integrative biology arena [55,56].

From a clinical point of view, six different simulation experiments have to be developed from *In Silico* Oncology. These models should answer the following questions for an individual patient [57]:

1. What is the natural local tumour growth over time in size and shape?
2. When and whereto is a tumour metastasising?
3. Can the response of the local tumour and the metastases to a given treatment be predicted in size and shape over time?
4. What is the best treatment schedule for a patient regarding drugs, surgery, irradiation and their combination, dosage, time schedule and duration?
5. Is it possible to predict severe adverse events (SAE) of a treatment and to propose an alternative treatment to avoid them without deteriorating outcome?
6. Is it possible to predict a cancer before it occurs and to recommend a treatment that will prevent the occurrence or a recurrence of a cancer in an individual patient?

The aim to develop an ‘oncosimulator’ within **ACGT** is to evaluate the reliability of *in silico* modelling as a tool. *In silico* oncology always has to be tested in the setting of clinico-genomic trials to prove the expectations for getting better individualized cancer treatments with higher cure rates and less acute and late toxicity. *In silico* oncology using and combining clinical imaging and genomic/genetic data will give doctors a better way to tailor cancer treatment; thus holding the promise of applying a more individualized treatment with increasing survival, reducing side effects and improving the quality of life. Additionally, it is a platform for better understanding and exploring the natural phenomenon of cancer, as well as training doctors and interested patients alike.

Although most patients with cancer respond to therapy, not all of these are cured. Even objective clinical responses to a given treatment do not translate into substantial improvements in overall survival. The reason for this phenomenon can be explained by the fact that therapies successfully eliminating the vast majority of cancer cells may be ineffective against rare biologically distinct cancer stem cells. Therefore, new methods for assessing treatment efficacy have to be developed as a traditional response criteria, such as the RECIST criteria, and their further developments [58, 59, 60] measure tumour bulk do not reflect changes in the rare cancer stem cells [61]. It seems obvious that treatment effective against the gross majority of differentiated cancer cells is ineffective for rare cancer stem cells. This suggests that treatment should be changed when a patient is in clinical remission, following the destruction or removal of the bulky tumour burden. *In silico* experiments should focus on this topic. Data on cancer stem cells for each tumour have to be created by molecular biologists, and clinicians have to provide them with tumour material. This again underlines the importance of enrolling patients into clinico-genomic trials if *in silico* experiments are carried out and conclusive results are awaited.

In order to achieve all of these goals, *in silico* oncology has to undergo a thorough clinical optimization and validation process. Nephroblastoma and breast cancer have been discussed to serve as two paradigms to clinically specify and evaluate the ‘oncosimulator’ as well as the emerging domain of *in silico* oncology.

**Figure 3:**
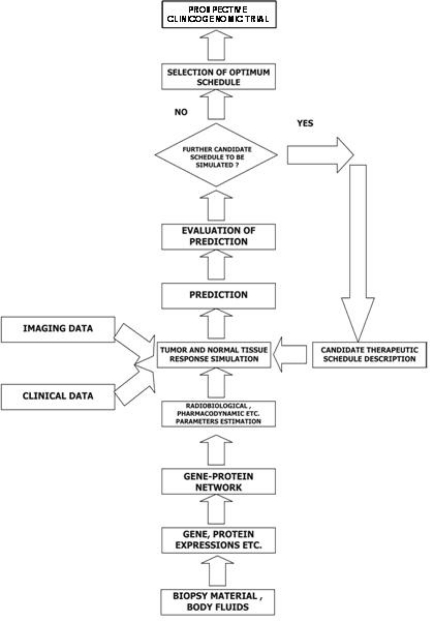
A block diagram of the oncosimulator’s function

The ‘oncosimulator’ is based on the ‘top-down’ multi-scale simulation strategy developed by the In Silico Oncology Group National Technical University of Athens (www.in-silico-oncology.iccs.ntua.gr) [62–65]. The imaging histopathological molecular and clinical data of any given patient following pertinent pre-processing are introduced into the Tumour and Normal Tissue Response Simulation Module, which executes the simulation code for a defined candidate treatment scheme (Figure 3). The prediction is judged by the clinician, and further schemas can be done in an analogous way. Finally, the clinician decides on the optimal treatment scheme to be administered to the patient based on his or her formal medical education and knowledge and the predictions of the ‘oncosimulator’ after retrospective and prospective validation.

## Legal and ethical aspects

In the context of medical research involving patients, the ethical principle of autonomy is generally recognized as one of the most basic principles. Derived from autonomy, the doctrine of informed consent has been widely acknowledged [66,67]. However, clinico-genetic research addresses new questions because data are collected and used not only for specific research questions but also for future research projects, which cannot be defined at the time consent is requested [68]. Furthermore, research results may be obtained, which could be important for individual patients or groups of individuals (e.g. family members). Facing these new demands doubts have been raised concerning the applicability of the doctrine of informed consent in its current form.

Research projects can only succeed if it is possible to create a framework that takes into account the needs of modern scientific genetic research and the needs of the patients regarding data protection and privacy. Only if these two conditions are met can such research projects succeed. In ACGT, participants will be provided with adequate and understandable information regarding data sampling, storage and usage. The given information for informed consent must always include:

1. the main intentions of ACGT;
2. the voluntariness of participation in the research;
3. the range of how data are used;
4. the measures that are taken to protect the personal rights of donors;
5. the possible risks and benefits of the research;
6. further implications of participation.

In ACGT, a tiered consent will be used referring to clinico-genomic research on cancer in the context of the specific structure of the project. Informed consent is necessary for patients participating in ACGT trials and for authorized users of the ACGT grid structure before getting access. They have to declare that they will meet the requested standards of ACGT regarding the protection of data and privacy.

Since clinico-genomic research may yield individually important research results, the question of whether and under what circumstances data should or must be fed back to the patients concerned has to be discussed. It is widely acknowledged that general study findings must be accessible for patients involved [69,70]. Furthermore, anybody has the right to access personal data stored about him or her. But the right to access such data, which is based on ethical principles as well as on legal provision, is a passive one. Therefore, the implementation of this right requires an organizational structure that is suitable to reply to donors’ requests. Additionally, it is recommended that ACGT provides the technical and organizational means for individual feedback processes of such results initiated by the investigator. The only way to enable investigator-driven individual feedback processes—and to allow individual donors to withdraw consent—is the pseudonymization of data. Therefore, the process of feeding back individually relevant data requires technical mechanisms to guarantee data retrieval by those donors who ask for individual feedback. Nevertheless, the discussion what kind of data can be fed back is controversial, since the relevance of data is not easy to define [71,72]. From an ethical point of view, it is therefore recommended to give the patients the option to decide about feedback of personal data and allow them to withdraw their consent. Every individual feedback process should also be accompanied by consultation. Given the complexity of the ethical aspects regarding the disclosure and feedback, a multi-lingual internet-based information service for donors will be established within ACGT.

As genetic data are very sensitive data, which hold information not only about the data subject itself but also about his or her relatives’ possible diseases, etc, the processing of this kind of data is only possible under special requirements. Genetic data are also very vulnerable and can only be de-facto anonymized, which means that—at least in theory—a re-identification is always possible if matching information from the genetic code of that of a known person. This is the big difference to normal conventional data and a challenge for the application of data protection regulation.

The data protection structure to be established for ACGT has to find a balance for the two competing aims of modern genetic research and the data protection needs of the participating patients. In order to comply with current data protection legislation, it is recommended that as much of the patient’s genetic data as possible is (de-facto) anonymized. As long as there is no link between de-facto anonymized genetic data and the data subject, they can be regarded as anonymous and can be kept outside of the scope of the Data Protection Directive 95/46/EC [73]. Following that the Data Protection Directive is applicable whenever the particular data controller has the link from the genetic data to the concerned data subject or whenever he can get this link with legal means or whenever a third party can establish this link. Therefore, the genetic data have to be regarded as personal data in the case of transfer and disclosure. In all other cases of data processing, for example usage and storage, the Data Protection Directive is not applicable as long as the data controller has no legal access to the link. Besides that, an informed consent of the participating patients is needed because of ethical reasons and as a fallback solution for the legal data protection framework [74].

Furthermore, a data protection framework has to be set up for ACGT, which consists mainly of three parts. First, an ACGT Data Protection Board has to be implemented. It will be the central data controller within ACGT as well as a legal body able to conduct contracts regarding data protection on behalf of ACGT. Second, a Trusted Third Party is needed in this data protection framework, which is responsible for the pseudonymization of the patient’s genetic data, and which will also be the keeper of the pseudonymization key to re-identify the patient concerned. Therefore, the patient’s genetic data is de-facto anonymous for users and participants of ACGT not having the link. Third, contracts between all participating hospitals research units or other users of the genetic data and ACGT must be concluded in order to ensure confidentiality data security and compliance with data protection legislation.

By implementing this framework, the needs of the researchers hospitals and patients can be satisfied at the same time so that the **ACGT** Data Protection Framework is a milestone to lead **ACGT** to success. It allows participating researchers to concentrate on their scientific research without dealing with data protection issues.

## Summary

During the last few years, the ‘omics’ revolution has dramatically increased the amount of data available for characterizing intracellular events. As a result, a lot of patterns of gene expression were found that could be used to classify molecular subtypes of tumours and predict the outcome and response to treatment. Currently, the main focus is on interlinking the various data sources generated by high-throughput array technologies. Various groups have applied network analysis to gene data sets associated with cancer. **ACGT**, a project funded by the European Commission in the Sixth Framework Programme, goes far beyond these networks by the integration of clinical data. The ultimate objective of the **ACGT** project is the provision of a unified technological infrastructure, which will facilitate the seamless and secure access and analysis of multi-level clinical and genomic data enriched with high-performing knowledge discovery operations and services. By doing so, it is expected that the influence of genetic variation in oncogenesis will be revealed, the molecular classification of cancer and the development of individualized therapies will be promoted, and finally, the *in silico* tumour growth and therapy response will be realistically and reliably modelled. Achieving these goals, **ACGT** will not only secure the advancement of clinico-genomic trials, but will also achieve an expandable environment to other studies’ technologies and tools.

Today, it is recognized that the key to individualizing treatment for cancer lies in finding a way to quickly ‘translate’ the discoveries about human genetics made by laboratory scientists into tools that physicians can use in making decisions about the best way to treat patients. This area of medicine that links basic laboratory study to clinical data, including the treatment of patients, is called translational research and is promoted by clinico-genomic trials running in ACGT. These clinico-genomic trials are scenario based and driven by clinicians. Today, two main clinico-genomic trials and an *in silico* experiment are interconnected within the ACGT project. The realization of these trials will act as benchmark references for the development and assessment of the ACGT technology.

All ethical and legal requirements for clinico-genomic trials will be respected. A data protection framework will be set up for ACGT, which consists of an ACGT Data Protection Board, a Trusted Third Party responsible for the pseudonymization of the patient’s data and contracts between all participating hospitals research units or other users of genetic data.

Patients who take part in clinico-genomic trials may be helped personally by the treatment(s) they receive. They get up-to-date care from cancer experts, and they receive either a new treatment being tested or the best available standard treatment for their cancer. Of course, there is no guarantee that a new treatment being tested or a standard treatment will cure the patient. New treatments also may have unknown risks, but if a new treatment proves effective or more effective than standard treatment trial patients who receive it may be among the first to benefit.

## Figures and Tables

**Table 1:**
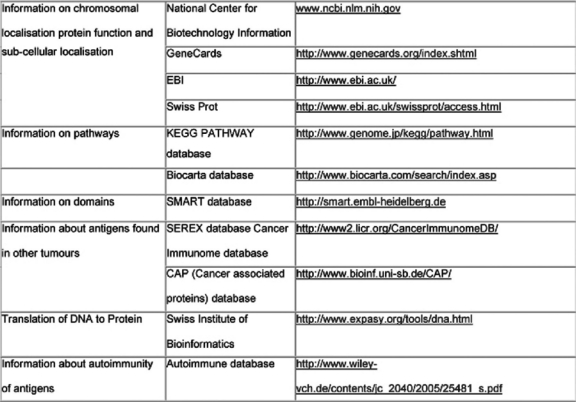
Data available from websites
